# Clinical‐radiological approach for the diagnosis of cleidocranial dysplasia in adults: A familial cases series

**DOI:** 10.1002/ccr3.5235

**Published:** 2021-12-26

**Authors:** Javier Ignacio Segovia‐Fuentes, Jorge Armando Egurrola‐Pedraza, Edgar Junior Castro‐Mendoza, Eder Cano‐Pérez, Doris Esther Gómez‐Camargo, Dacia Isabel Malambo‐García

**Affiliations:** ^1^ Facultad de Medicina Departamento de Diagnóstico Universidad de Cartagena Cartagena de Indias Colombia; ^2^ Facultad de Ciencias de la Salud Universidad del Magdalena Santa Marta Colombia; ^3^ Grupo de Investigación UNIMOL Facultad de Medicina Universidad de Cartagena Cartagena de Indias Colombia; ^4^ Facultad de Medicina Doctorado en Medicina Tropical Universidad de Cartagena Cartagena de Indias Colombia

**Keywords:** bone disease, cleidocranial dysplasia, orphan disease, rare disease

## Abstract

Cleidocranial dysplasia is a rare disease with an autosomal‐dominant inheritance that mainly affects the bones of the axial skeleton. In this report, we discuss the clinical and radiological signs of a case series comprising three sisters and the son of one of the sisters, all with suspected bone dysplasia.

## Graphical abstract

The reported case series, to our knowledge, is the first family case of CCD in Colombia. It is important to take timely and appropriate action on disorders of genetic origin that can lead to deterioration in the quality of life of patients.

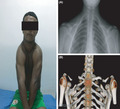

## 1 INTRODUCTION

Cleidocranial dysplasia (CCD, OMIM #119600)^1^ is a genetic disease that compromises general bone development, presenting open cranial sutures with bulging of the frontal and parietal bones, hypoplasia or aplasia of the clavicles, and maxillary alterations with delays in changes of decidual teeth and supernumerary permanent teeth; brachydactyly and hypoplastic distal phalanges in the hands; and hypoplasia of the pelvis with wide pubic symphysis.^2^, ^3^ CCD is considered a rare or orphan disease (ORPHA: 1452) within the group of primary bone dysplasias, with an estimated prevalence of 1–9 cases per 1,000,000 population (www.orpha.net).

The disease is inherited in an autosomal‐dominant manner and presents with complete penetrance and variable clinical expression of the phenotype in family groups. However, de novo cases have been described with frequencies of up to 40% in some populations, and some cases with an autosomal‐recessive inheritance pattern have been reported.^4^, ^5^ CCD is caused by mutations in the *RUNX2*/*CBFA1* gene located at the 6p21 locus, which encodes a transcription factor that activates osteoblastic differentiation.^6^, ^7^ Various nonsense, antisense, and frameshift mutations have been identified that cause haploinsufficiency in the CBFA1 protein, and translocations and chromosomal deletions that lead to the loss of the complete gene.^7^, ^8^, ^9^

The presence of the three pathognomonic clinical signs of CCD, including cranial, maxillary, and clavicle morphological alterations, provides relevant information for the diagnosis of the disease. However, other skeletal dysplasias share characteristics with CCD.^3^ Therefore, to confirm the clinical diagnosis, imaging studies such as skull, chest, spine, pelvis, hands, and feet radiographs, and panoramic radiography, are important and highly required.^2^, ^3^

The early diagnosis of CCD is essential to promptly treat the complications of the disease through a multidisciplinary health team. The condition in patients is commonly identified during childhood or adolescence; however, diagnosis may be delayed until adulthood if the symptoms and signs are not severe.^10^ In this report, cases of four relatives with a clinical‐radiological diagnosis of cleidocranial dysplasia in adulthood are described. Patients were referred to the UNIMOL research group, University of Cartagena, for suspected genetic disease. This report was prepared following the recommendations of the CARE guide.^11^

## 2 CASE PRESENTATIONS

### 2.1 Case 1

**FIGURE 1:**
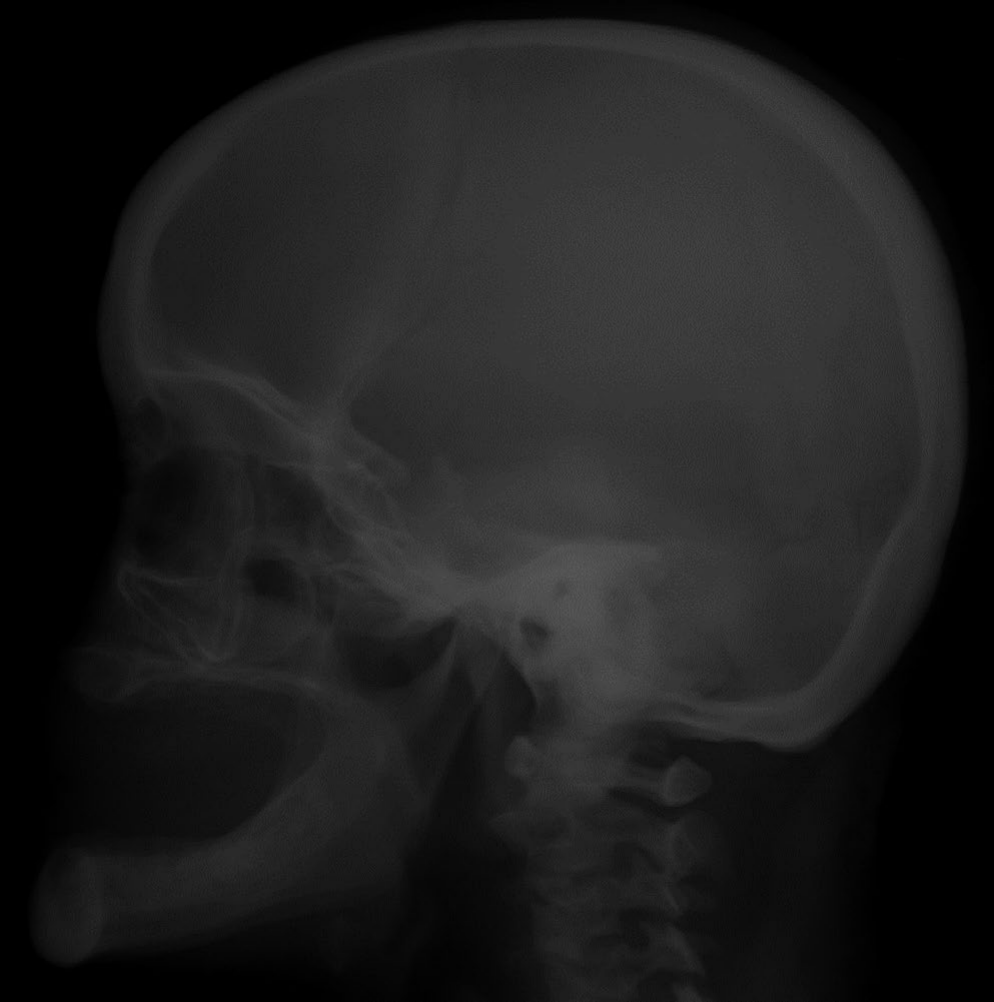
Simple skull X‐ray of case 1 showing prominent chin with underdevelopment of the maxillary bone, relative prognathism (pseudoprognathism), and the absence of dental pieces

A 33‐year‐old woman requested guidance from the genetics service center at the UNIMOL group. During the initial examination, she was referred for dental treatment due to poor dental health and dental pain, which had affected her social relationships. She was subjected to extractions of the remaining teeth and dental prosthesis adaptation. In addition, the clinical findings revealed brachycephaly, frontal and parietal bulging, no open fontanelles, hypertelorism, depressed nasal bridge, oral cavity with an absence of teeth, micrognathia and prognathism, short neck, clavicles that were not palpable, and shoulders that approached the midline. Skull X‐ray revealed a prominent chin due to an underdeveloped maxilla, a relatively prognathic mandible (pseudoprognathism), and the absence of dentition in the maxillae (Figure 1). Chest radiography revealed hypoplasia of the right clavicle and bell‐shaped chest. No alterations in the spine, pelvis, or hip were evident during the physical examination. Based on the clinical and radiological findings, the diagnosis of cleidocranial dysplasia was confirmed. Regarding her family history, the patient manifested that some members of her family presented similar clinical conditions, which made it possible to assess her two sisters (cases 2 and 3) and a nephew (case 4).

### 2.2 Case 2

**FIGURE 2:**
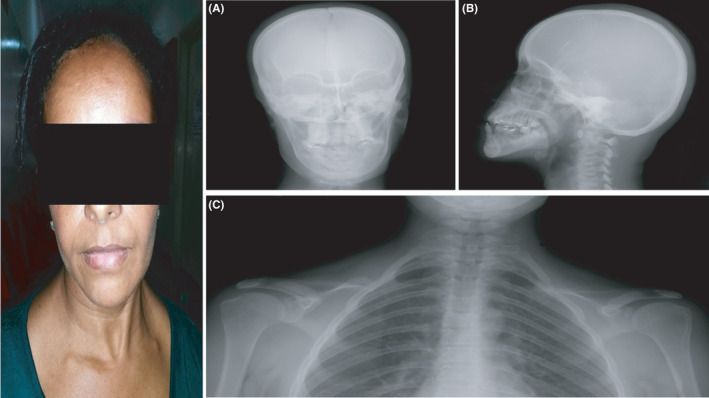
X‐ray studies of the skull (A, B) and chest (C) showing slight separation of the sagittal suture, retention of teeth, hypoplasia of the clavicles, and a bell‐shaped chest

The patient was a 40‐year‐old woman whose physical examination revealed mild brachycephaly, symmetric and biparietal frontal bulge, hypertelorism, depressed nasal bridge, prognathism, micrognathia, oral cavity with damaged and absent molars, and hypoplastic clavicles to the touch with shoulders that approached the midline; no metopic ridge was observed, and open fontanelles were not palpated. Short thumbs and flat feet were observed. X‐ray studies of the skull and chest showed slight separation of the sagittal suture, retention of teeth, hypoplasia of the clavicles, and a bell‐shaped chest (Figure 2). No alterations in the spine, pelvis, or hip were evident during the physical examination.

### 2.3 Case 3

**FIGURE 3:**
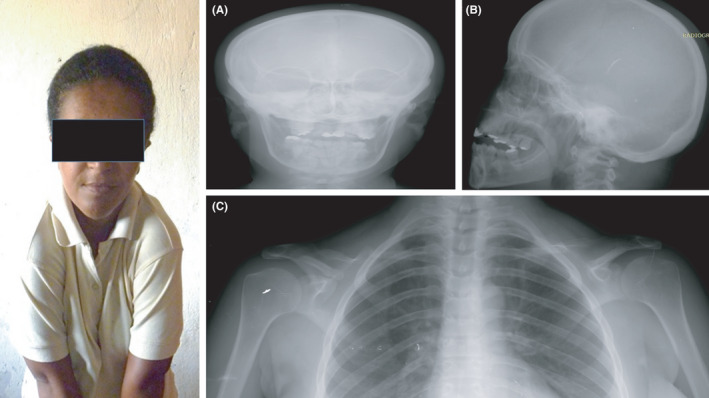
X‐ray studies of the skull (A, B) and chest (C) showing retention of teeth in the upper and lower jaw, bell‐shaped chest, the absence of the distal ends of both clavicles, and hypoplasia of the middle thirds more pronounced in the right clavicle

During medical consultation, a 45‐year‐old woman reported that she had moderate pain in her left shoulder for approximately 6 months, which was exacerbated by physical activity and improved upon using analgesics. Clinically, brachycephaly, symmetric and biparietal frontal bulging, hypoplasia in the middle part of the face, prognathism, micrognathia, hypertelorism, and depressed nasal bridge were observed. No open fontanelles were palpated, and no metopic crest was observed. The patient presented with an oral cavity with damaged molars and some absent teeth, short neck, limited range of motion in the left shoulder, and hypoplastic clavicles at the touch with shoulders that approached the midline. Flat feet and hands with short fingers were observed, mainly the thumb of both hands. X‐ray studies of the skull and chest showed retention of teeth in the upper and lower jaw, bell‐shaped chest, absence of the distal ends of both clavicles, and hypoplasia of the middle thirds more pronounced in the right clavicle (Figure 3). No alterations in the spine, pelvis, or hip were evident during the physical examination.

### 2.4 Case 4

**FIGURE 4:**
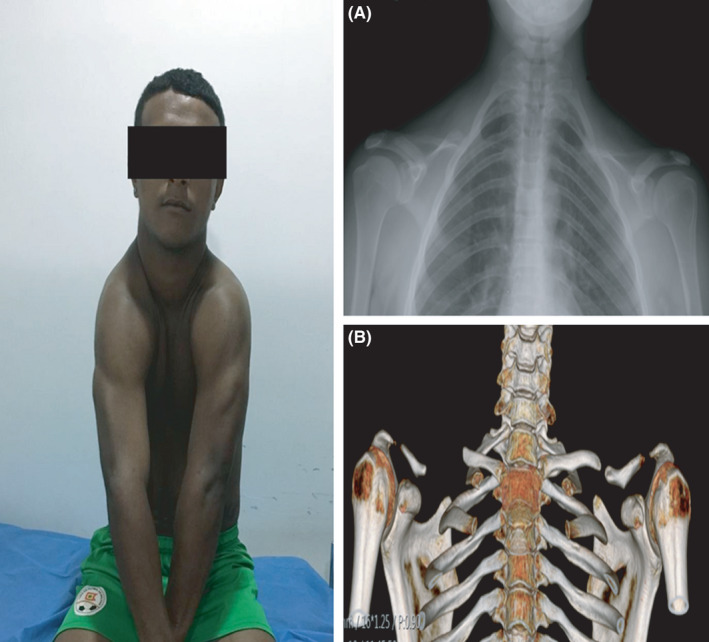
X‐ray (A) and tomography with three‐dimensional reconstruction of the chest (B) of case 4 showing a bell‐shaped thorax and hypoplastic clavicles with fusion defects toward the middle thirds

**FIGURE 5:**
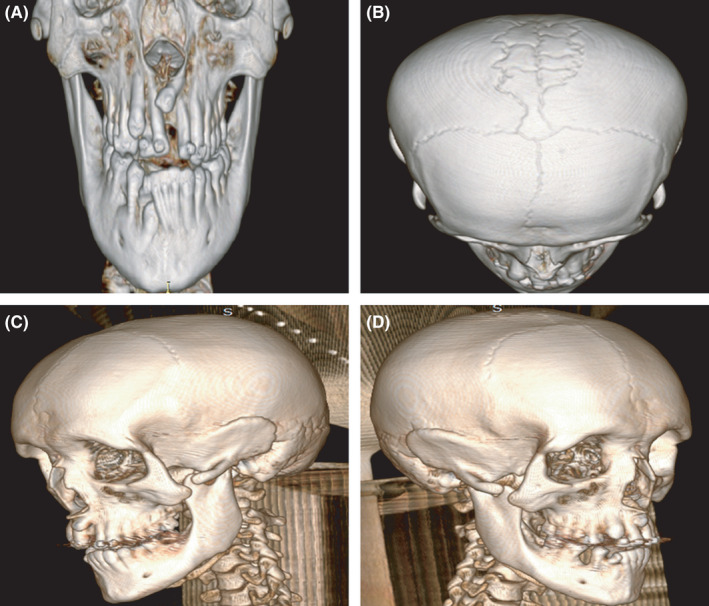
Tomography with three‐dimensional reconstruction of the skull and face of case 4 showing (A) nonerupted teeth in the upper and lower dental arch and crowding of teeth in the lower dental arch, (B) multiple Wormian bones in the vicinity of the sagittal suture, and (C‐D) bone defects in both zygomatic arches that do not articulate with malar bone

The patient was a 21‐year‐old man and son of patient 3. During the consultation, the patient presented with musculoskeletal pain in the upper limbs, which focused on the bilateral acromioclavicular joints. In the clinical findings, brachycephaly, frontal and parietal bulging, hypertelorism, micrognathia, prognathism, and crowding of teeth in the upper jaw were evidenced. On inspection of the thorax, clavicle hypoplasias at the touch with shoulders that approached the midline were observed. In addition to the X‐ray, the patient underwent computerized axial tomography of the head and hemithorax with volumetric acquisition techniques and three‐dimensional reconstructions (Figures 4 and 5). The findings on the face were consistent with the lack of fusion of the zygomatic arches in the anterior third, supernumerary, and nonerupted teeth in the upper dental arch. In the skull, numerous Wormian bones were found near the sagittal and lamboid sutures. Finally, in the upper thorax, a hypoplastic right clavicle was observed with the absence of a large part of the middle third, while the left clavicle presented a lesser degree of hypoplasia with the absence of a part of the external third. The X‐ray showed a bell‐shaped chest. No alterations in the spine, pelvis, or hip were evident during the physical examination.

## 3 DISCUSSION

The clinical and radiological approach applied to the cases was conclusive for the diagnosis of CCD due to the identification of several pathognomonic clinical signs of the disease. In general, the diagnosis of CCD is made in childhood or adolescence; however, four adult cases are described in this report. Case 1 requested guidance for genetics, and as a benefit, it was possible to provide dental treatment that improved their appearance and social relationships. This advance allowed the patient to refer her two sisters and a nephew for medical evaluation.

CCD presents clinical signs of dysmorphology of bones located in the skull, maxillae, and thorax; these signs are pathognomonic for the disease.^2^, ^3^, ^12^ The most frequent radiological findings in the skull are multiple Wormian bones, segmental thickening of the calvaria, lack of ossification of the sutures, persistent fontanelles, dysplastic changes in the occiput, hypoplasia of the maxilla, absence or delayed mineralization of the nasal bones, and hypoplastic sinuses.^3^, ^12^, ^13^, ^14^, ^15^ Supernumerary and impacted teeth are common in the maxilla.^16^ In the chest, hypoplasia/aplasia or discontinuous clavicles, bell‐shaped chest, absence of ribs, and hypoplasia of the scapulae are common.^12^, ^13^, ^17^ Other conditions include cervical and lumbar scoliosis and pelvic bone anomalies.^3^, ^18^ Finally, in radiographs of the hands, middle phalanges, metacarpals, and tarsi short, hypoplastic distal phalanges, accessory epiphysis, and cone shape can be found.^2^, ^19^, ^20^Previously described skeletal alterations are caused by mutations in the *RUNX2*/*CBFA1* gene.^7^The inheritance mechanism is dominant with full penetrance; expressivity is variable and is evidenced in the different clinical spectra observed in the case series, ranging from classic phenotypes to severe cases with a total absence of parietal bones.^21^, ^22^ The spectrum of mutations is variable, with findings of deletions, inversions, and insertions that generate nonsense, antisense, and frameshift mutations previously reported.^4^, ^12^, ^23^

The cases described in this study have been added to the few reports made in Colombia regarding this disease.^24^, ^25^, ^26^, ^27^, ^28^ For reference, Medina et al.^24^and Ortega and Suárez^25^ described two pediatric cases of 3 and 6 years old, respectively. In both cases, short stature was the reason for medical consultation, and during physical examinations, late closure of fontanelles and some skeletal alterations were the main suspicion of CCD, which was confirmed by subsequent radiological studies and molecular studies in one of the cases. Conversely, Harris et al.^26^, ^27^ reported two cases of adolescents aged 12 and 16 years who attended a dental consultation due to delayed secondary dentition eruption. In addition to the dental signs, physical examinations and radiological findings led to the diagnosis of CCD. As in the previous cases, the diagnosis is regularly made in childhood or adolescence.^17^, ^29^ However, we report a case series of CCD diagnosed in adult individuals between 21 and 45 years old belonging to a family, except case 4, which was reported in childhood by Harris et al.^27^and whose clinical study has been expanded in adulthood in this report.

Clinical findings of our cases were similar to those reported by Gomleksiz et al.^10^in a 24‐year‐old man with alterations in the skull, dentition, and clavicles. However, this case presented delayed closure of the anterior fontanel, hearing loss, rhinolalia, dyspnea, and fatigue. In another report of a 22‐year‐old woman, cranial alterations with Wormian bones and wide sutures, loss of teeth, persistent decidual teeth, supernumerary teeth as revealed by pantomography, and hypoplastic right clavicle were detected. In addition, the findings of a polycystic ovary, bicornuate uterus, and Mullerian alterations were described.^30^In a report of a mother and her two sons, a 28‐year‐old woman had a wide anterior fontanelle, hypertelorism, drooping shoulders, distal finger phalanges, and hypoplastic clavicles; both the skull and chest X‐rays confirmed the findings of open anterior fontanel and hypoplastic clavicles.^31^An important aspect of the diagnosis of CCD in adult patients is the possible secondary complications, such as coxa vara, infections in the auditory system, and difficulties in vaginal delivery, for which cesarean sections are required.^32^, ^33^ Kobayashi et al.^34^reported a case of atlantoaxial subluxation‐induced myelopathy spastic in a 27‐year‐old patient with a history of CCD; the treatment involved surgery with cervical laminectomy. Furthermore, Vakili and Jalali^35^reported a case of hypogonadotropic hypogonadism associated with CCD in an adolescent patient. Regarding pediatric case reports, the same clinical signs described in adult patients are generally found.^24^, ^25^, ^28^, ^31^, ^36^, ^37^Other disorders share characteristics with CCD such as Crane–Heise syndrome, mandibuloacral dysplasia, pycnodysostosis, Yunis–Varon syndrome, CDAGS syndrome, and hypophosphatasia among others. However, these disorders have other clinical and bone characteristics specifically different from CCD.^3^ In all cases, bone images play a distinctive role in the differential diagnosis of skeletal dysplasias.^38^ Therefore, a complete skeletal study of the whole body that includes orthogonal views of the skull, spine, pelvis, and all limbs with separate views for the hands is recommended.^3^, ^38^CCD complications do not present a curative therapy; however, some of the disease conditions can be corrected through multidisciplinary treatment aimed at improving the health, esthetics, and quality of life of patients.^39^ Dental complications are usually the main intervened sign, and treatment generally involves exodontic and orthodontic procedures; however, the management of complications is challenging and in the long term that requires careful planning.^3^, ^13^ On the other hand, some other surgical procedures to correct cranial bone insufficiency have been performed.^40^, ^41^ It is important to act appropriately on disorders that chronically occur and that could lead to a deterioration in the self‐esteem and quality of life of patients; in all cases, timely recognition of the disease is vital for better management of complications through a multidisciplinary health team.

In conclusion, CCD should be suspected in patients with abnormal skull bones, clavicles, and teeth development. Early diagnosis is important to act appropriately on those disorders. Therefore, diagnostic support from the radiologist is important to characterize the malformations that require treatment to avoid complications and disability. An accurate clinical and radiological examination of CCD is important, especially when genetic testing is not performed or is not available. However, mutation analysis of the RUNX2 gene is recommended in cases requiring molecular confirmation. In future studies, we expect to identify the disease‐causing mutation in the *RUNX2*/*CBFA1* gene using molecular tests to support the clinical diagnosis of the individuals presented here. Colombian legislation protects people with orphan diseases and their families (Law 1392 of 2010 and Law 1438 of 2011), of which genetic diseases are the majority. Thus, in 2018, resolution 005265 of the Ministry of Health and Social Protection was created, which facilitated the updating of the list of orphan diseases and defined the number with which each of them is identified. This list is necessary for the provision of health services to people affected by these diseases. However, CCD, of which four cases have been reported here, has not yet been included in the list, a task to be performed in the next update.

## CONFLICTS OF INTEREST

The authors have no conflicts of interest to declare.

## AUTHOR CONTRIBUTIONS

JSF, JEP, ECM, and DMG involved in patient management and drafted the manuscript. DMG provided the images. ECP, DGC, and DMG edited and reviewed the article. All authors approved the final version of the manuscript.

## CONSENT

The patients signed the informed consent, which explained the use of the information from the clinical history and photographic material, for biomedical research purposes and divulgation in scientific media.

## Data Availability

All data used to support our findings are included in the manuscript. Given the nature of the study, the corresponding author may provide any additional information that does not compromise the confidentiality of the participating individuals upon request.

## References

[1] McKusick V , O´Neill M . Cleidocranial dysplasia. OMIM; 2013.

[2] Mundlos S . Cleidocranial dysplasia: clinical and molecular genetics. J Med Genet. 1999;36:177‐182. 10204840 PMC1734317

[3] Machol K , Mendoza‐Londono R , Lee B . Cleidocranial Dysplasia Spectrum Disorder. 2006 Jan 3 [Updated 2017 Nov 16]. In: Adam MP , Ardinger HH , Pagon RA et al., editors. GeneReviews® [Internet]. University of Washington, Seattle; 1993‐2021. https://www.ncbi.nlm.nih.gov/books/NBK1513/ 20301686

[4] Lee K‐E , Seymen F , Ko J , et al. RUNX2 mutations in cleidocranial dysplasia. Genet Mol Res. 2013;12(4):4567‐4574. 24222232 10.4238/2013.October.15.5

[5] Goodman RM , Tadmor R , Zaritsky A , Becker SA . Evidence for an autosomal recessive form of cleidocranial dysostosis. Clin Genet. 1975;8:20‐29. 1149318 10.1111/j.1399-0004.1975.tb01950.x

[6] Bergwitz C , Prochnau A , Mayr B , et al. Identification of novel CBFA1 / RUNX2 mutations causing cleidocranial dysplasia. J Inherit Metab Dis. 2001;24:648‐656. 11768584 10.1023/a:1012758925617

[7] Mundlos S , Otto F , Mundlos C , et al. Mutations involving the transcription factor CBFA1 cause cleidocranial dysplasia. Cell. 1997;89:773‐779. 9182765 10.1016/s0092-8674(00)80260-3

[8] Otto F , Kanegane H , Mundlos S . Mutations in the RUNX2 gene in patients with cleidocranial dysplasia. Hum Mutat. 2002;19:209‐216. 11857736 10.1002/humu.10043

[9] Quack I , Vonderstrass B , Stock M , et al. Mutation analysis of core binding factor A1 in patients with cleidocranial dysplasia. Am J Hum Genet. 1999;65(5):1268‐1278. 10521292 10.1086/302622 PMC1288279

[10] Gömleksiz C , Arslan E , Arslan S , Pusat S , Arslan EA . Delayed diagnosis of cleidocranial dysplasia in an adult: a case report. Acta Med Acad. 2014;43(1):92‐96. 24893645 10.5644/ama2006-124.106

[11] Riley DS , Barber MS , Kienle GS , et al. CARE guidelines for case reports: explanation and elaboration document. J Clin Epidemiol. 2017;89:218‐235. 28529185 10.1016/j.jclinepi.2017.04.026

[12] Motaei J , Salmaninejad A , Jamali E , et al. Molecular genetics of cleidocranial dysplasia. Fetal Pediatr Pathol. 2021;40(5):442‐454. 31984822 10.1080/15513815.2019.1710792

[13] Farrow E , Nicot R , Wiss A , Laborde A , Ferri J . Cleidocranial dysplasia: a review of clinical, radiological, genetic implications and a guidelines proposal. J Craniofac Surg. 2018;29(2):382‐389. 29189406 10.1097/SCS.0000000000004200

[14] Azevedo Almeida LC , Faraj de Lima FB , Matushita H , Valença MM , Ferreira Castro TL , de Mendonça RN . Cleidocranial dysplasia, a rare skeletal disorder with failure of the cranial closure: case‐based update. Childs Nerv Syst. 2020;36(12):2913‐2918. 32734401 10.1007/s00381-020-04831-z

[15] Pan C‐Y , Tseng Y‐C , Lan T‐H , Chang H‐P . Craniofacial features of cleidocranial dysplasia. J Dent Sci. 2017;12(4):313‐318. 30895069 10.1016/j.jds.2017.07.002 PMC6395362

[16] Symkhampha K , Ahn GS , Huh K‐H , Heo M‐S , Lee S‐S , Kim J‐E . Radiographic features of cleidocranial dysplasia on panoramic radiographs. Imaging Sci Dent. 2021;51(3):271‐278. 34621654 10.5624/isd.20201007 PMC8479436

[17] Jirapinyo C , Deraje V , Huang G , Gue S , Anderson PJ , Moore MH . Cleidocranial dysplasia: management of the multiple craniofacial and skeletal anomalies. J Craniofac Surg. 2020;31(4):908‐911. 32224772 10.1097/SCS.0000000000006306

[18] Berkay EG , Elkanova L , Kalaycı T , et al. Skeletal and molecular findings in 51 cleidocranial dysplasia patients from Turkey. Am J Med Genet A. 2021;185(8):2488‐2495. 33987976 10.1002/ajmg.a.62261

[19] Tanaka JLO , Ono E , Filho EM , Castilho JCM , Moraes LC , Moraes MEL . Cleidocranial dysplasia: importance of radiographic images in diagnosis of the condition. J Oral Sci. 2006;48(3):161‐166. 17023750 10.2334/josnusd.48.161

[20] Castrillón GA , Osorio ML , Serrano PEG , Rengifo DN . Hallazgos imagenológicos de la displasia cleidocraneal. Rev Argentina Radiol. 2017;81(3):229‐231.

[21] Bufalino A , Paranaíba L , Gouvêa AF , et al. Cleidocranial dysplasia: Oral features and genetic analysis of 11 patients. Oral Dis. 2012;18:184‐190. 22023169 10.1111/j.1601-0825.2011.01862.x

[22] Cunningham ML , Seto ML , Hing AV , Bull MJ , Hopkin RJ , Leppig KA . Cleidocranial dysplasia with severe parietal bone dysplasia: C‐terminal RUNX2 mutations. Birth Defects Res Part A ‐ Clin Mol Teratol. 2006;76:78‐85. 10.1002/bdra.20231 16463420

[23] Ott CE , Leschik G , Trotier F , et al. Deletions of the RUNX2 gene are present in about 10% of individuals with cleidocranial dysplasia. Hum Mutat. 2010;31:1587‐1593. 10.1002/humu.21298 20648631

[24] Medina O , Muñoz N , Moneriz C , Pretell CM . Displasia cleidocraneal: reporte de un caso. Rev Chil Pediatr. 2017;88(4):517‐523. 28898321 10.4067/S0370-41062017000400012

[25] Ortega RI , Suárez OF . Displasia cleidocraneal: presentación de un caso. Univ Médica. 2016;57(1):115‐122.

[26] Harris J , Álvarez L , Díaz A . Parámetros diagnósticos de la displasia cleidocraneal: una enfermedad poco frecuente. Arch Med. 2017;17(2):2339‐3874.

[27] Harris Ricardo J , Rebolledo Cobos M , Fortich MN . Hiperodoncia múltiple y su relación con la displasia cleidocraneal. Av Odontoestomatol. 2011;29(1):25‐29.

[28] Castrillón GA , Osorio ML , Gil‐Serrano PE , Noreña‐Rengifo D . Hallazgos imagenológicos de la displasia cleidocraneal. Rev Argent Radiol. 2017;81(3):229‐231.

[29] Ramos‐Mejía R , Rodríguez‐Celin M , Fano V . Clinical, radiological, and auxological characteristics of patients with cleidocranial dysplasia followed in a pediatric referral hospital in Argentina. Arch Argent Pediatr. 2018;116(4):e560‐e566. 30016033 10.5546/aap.2018.eng.e560

[30] Mohan VS , Desai RS , Patil MB . Cleidocranial dysplasia with bilateral polycystic ovarian disease and Mullerian abnormality of the uterus: A case report. J Oral Pathol Med. 2006;35:311‐113. 16630296 10.1111/j.1600-0714.2006.00399.x

[31] Pamuk ÖN , Mundlos S , Çakir N . Cleidocranial dysplasia in a mother and her two children. Jt Bone Spine. 2008;75:725‐727. 10.1016/j.jbspin.2007.10.013 18818114

[32] Currall V , Clancy R , Dimond D , Amirfeyz R , Kershaw C , Gargan M . Cleidocranial dysplasia. Curr Orthop. 2007;21(2):159‐162.

[33] Cooper S , Flaitz C , Johnston D , Lee B , Hecht J . A natural history of cleidocranial dysplasia. Am J Med Genet. 2001;104(1):1‐6. 11746020 10.1002/ajmg.10024

[34] Kobayashi S , Uchida K , Baba H , et al. Atlantoaxial subluxation–induced myelopathy in cleidocranial dysplasia. J Neurosurg Spine. 2007;7:243‐247. 17688067 10.3171/SPI-07/08/243

[35] Vakili R , Jalali F . Hypogonadotropic hypogonadism associated with cleidocranial dysostosis. J Pediatr Endocrinol Metab. 2005;18(9):917‐919. 16279370 10.1515/jpem.2005.18.9.917

[36] Karagüzel G , Aktürk FA , Okur E , Gümele HR , Gedik Y , Okten A . Cleidocranial dysplasia: a case report. J Clin Res Pediatr Endocrinol. 2010;2(3):134‐136. 21274329 10.4274/jcrpe.v2i3.134 PMC3005677

[37] Shen Z , Zou CC , Yang RW , Zhao ZY . Cleidocranial dysplasia: report of 3 cases and literature review. Clin Pediatr (Phila). 2009;48(2):194‐198. 18832541 10.1177/0009922808323107

[38] El‐Sobky TamerA , Shawky RabahM , Sakr HossamM , et al. A systematized approach to radiographic assessment of commonly seen genetic bone diseases in children: a pictorial review. J Musculoskelet Surg Res. 2017;2:25‐32.

[39] Chalala C , Noujeim ZEF , Abou Chebel N , Saadé A . Multidisciplinary management of cleidocranial dysplasia. J World Fed Orthod. 2015;4(1):31‐39.

[40] Hwang S‐M , Park B , Hwang M‐K , Kim M‐W , Lee J‐S . Aesthetic facial correction of cleidocranial dysplasia. Arch Craniofac Surg. 2016;17(2):82‐85. 28913260 10.7181/acfs.2016.17.2.82 PMC5556876

[41] Jung YT , Cho JI , Lee SP . Cranioplasty using a modified split calvarial graft technique in cleidocranial dysplasia. J Korean Neurosurg Soc. 2015;58(1):79‐82. 26279819 10.3340/jkns.2015.58.1.79 PMC4534745

